# Distinct Immunologic Properties of Soluble Versus Particulate Antigens

**DOI:** 10.3389/fimmu.2018.00598

**Published:** 2018-03-21

**Authors:** Clifford M. Snapper

**Affiliations:** ^1^Department of Pathology, Uniformed Services University of the Health Sciences, Bethesda, MD, United States

**Keywords:** bacteria, antibody, particle, vaccine, polysaccharide, autoantibody, marginal zone, antigen presentation

## Abstract

Antigens in particulate form have distinct immunologic properties relative to soluble antigens. An understanding of the mechanisms and functional consequences of the distinct immunologic pathways engaged by these different forms of antigen is particularly relevant to the design of vaccines. It is also relevant regarding the use of therapeutic human proteins in clinical medicine that have been shown to aggregate, and perhaps as a result, elicit autoantibodies.

## Differences Between Soluble and Particulate Antigens Relevant to the Immune Response

Relative to soluble antigens, antigens in particulate form are selectively internalized through antigen-presenting cell (APC) phagocytosis, with greater efficiency (1, 2) but with longer processing time (3), exhibit quantitative and qualitative differences in the antigenic epitopes generated (4), concentrate for extended periods within the marginal zone of the spleen (5), and are presented poorly, if at all, by splenic B cells (4), although efficiently internalized by peritoneal B1b cells (6). Signaling responses of APC can differ markedly in response to microbe-associated molecular patterns that are expressed in particulate versus soluble form (7). Various particulates, including intact bacteria activate the inflammasome resulting in production of IL-1β (8, 9), a cytokine that can augment T cell-dependent antibody responses (10). Phagocytosis of particulate antigens by APC also augment the calcineurin/NFAT signaling pathway resulting in a higher level of immune stimulation (7).

## Antigen Particulation Improves Vaccine Immunogenicity

The aggregation or particulation of an antigen could increase its overall immunogenicity by enhancing B cell receptor cross-linking, leading to higher levels of B cell activation and targeting of internalized antigen to lysosomes with subsequent enhanced antigen presentation to T cells. Thus, DNA vaccination with plasmids encoding for weakly immunogenic GFP protein fused to either a long polyQ domain that triggers aggregation or a short polyQ domain that does not resulted in a significantly higher anti-GFP antibody response to the GFP aggregate, relative to its non-aggregated form, as well as to enhanced CTL activity (11). The attachment of several vaccines to bacterium-like particles derived from the Gram-positive bacterium *Lactococcus lactis* that was treated to become a predominantly peptidoglycan shell (1–2 µm in diameter), has demonstrated significant enhancement in CD4^+^ T cell responses, and promotion of antigen cross-presentation for CD8^+^ T cell activation (12). Nanoparticles can also be used as a platform for synchronizing delivery of antigens and adjuvants that can be targeted to specific cell types (13). The particle size to which antigen is associated may play a critical factor in the subsequent immune response. Thus, intradermal immunization of mice with ovalbumin (OVA) covalently attached to a range of carboxylated polystyrene microspheres (0.02–2 µm in diameter) in the absence of adjuvant demonstrated the highest OVA-specific T cell and antibody responses when using 0.04 µm, but not larger, beads (14). The immune response using 0.04-µm beads was also higher relative to OVA immunizations using a number of different adjuvants. A subsequent study from this group further demonstrated a greater uptake by lung APC, and higher immune mediator release, following intratracheal instillation in mice of 0.05-µm, relative to 0.5-µm, diameter polystyrene nanoparticles (15). In this regard vaccines, in which recombinant proteins are displayed as virus-like particles, such as hepatitis B and human papilloma virus have proven clinically safe and highly effective in preventing the corresponding viral infections (16, 17). Many additional vaccination approaches using antigen particulation as a platform are currently under investigation (18–21), to mention only a few.

One underlying mechanism involved in the adjuvant effect of particulation is the targeted delivery of antigens to APCs in a concentrated form. We demonstrated that dendritic cells (DCs) were >5,000 times more efficient in the uptake and presentation of a bacterial protein to antigen-specific T-cells when delivered on the bacterial surface than when in soluble form, as a polysaccharide (PS)–protein conjugate (1). This likely reflected the fact that ingestion of a single bacterial particle by an APC effected the uptake of multiple copies of the associated protein. In contrast, the amount of soluble protein internalized *via* pinocytosis depended more heavily on the local concentration of antigen. Thus, the same total amount of soluble PS–protein conjugate delivered at a higher concentration was internalized 10–50 times more efficiently by the DC (1). PS–protein conjugates include several licensed clinical vaccines, such as that for *Haemophilus influenzae* type b, *Streptococcus pneumoniae*, and *Neisseria meningitidis*, that are highly protective against infections with these PS-encapsulated extracellular bacteria (22).

## Antigen Particulation Can Break Immune Tolerance

The immune system typically develops tolerance to self-proteins, yet autologous proteins used for therapeutic purposes often elicit antibody responses (23–25). Unwanted consequences of the latter include a reduction in drug efficacy (25) or development of significant pathologies (24, 26). Although the mechanism that underlies this break in immune tolerance is uncertain, possible contributors include degradation, modification, or aggregation of the protein (27, 28), or its contamination with Toll-like receptor (TLR) ligands (29, 30). In particular, aggregation has been implicated in immune responses to intravenous immunoglobulin, human growth hormone, and interferon α2 formulations (27, 28, 31, 32). Therapeutic proteins can aggregate in response to various stressors, such as agitation, freezing, and exposure to the air–liquid interface, during their manufacture, storage, and/or delivery to patients (33). Such aggregates may contain different secondary and tertiary structures that expose different epitopes, as well as create a repeating antigenic array for higher avidity B cell receptor binding and cross-linking. Indeed, immunization of rabbits or mice with virus-like particles to which arrays of self-antigens were conjugated induced strong antibody responses to those self-antigens (34, 35).

In light of the above, we directly tested the hypothesis that particulation of a soluble self-protein, i.e., mouse serum albumin (MSA), may lead to the breaking of self-tolerance in non-autoimmune mice, manifested by induction of CD4^+^ T cell-dependent antigen-specific antibody responses. This question was directly relevant to the fact that human serum albumin has a wide variety of clinical applications including intravascular volume expansion (36) and stabilization of protein therapeutics and vaccines (37). Certain properties of albumin would suggest a low likelihood of its eliciting autoantibodies. Thus, it exhibits limited polymorphism, including no known phenotypic variation in inbred mouse strains (38). In humans, although the gene for albumin is highly polymorphic, variations in the encoded protein sequences are rare (39). Moreover, during its synthesis, albumin is non-glycosylated, reducing its potential variability, although 6–15% may undergo nonenzymatic glycation in the blood (40, 41). Other properties of albumin, however, might suggest its potential for acting as an autoantigen. Thus, glycation alters the conformation and function of albumin (42). Albumin also binds various serum ligands (43) and interacts with a variety of host cells (44) and some bacterial pathogens (45–47). Bacteria can also bind albumin indirectly such as specific binding to heme that contains bound albumin (48).

In light of the potential for therapeutic proteins to aggregate as well as the observation that albumin can bind to intact bacterial surfaces, we wished to determine whether MSA covalently attached to bacteria-sized (1 µm) latex beads could induce an autoimmune response in non-autoimmune BALB/c mice. We observed that bead-associated, but not soluble MSA was indeed able to induce a CD4^+^ T cell-dependent MSA-specific IgG response (49). When MSA and PS (a T cell-independent antigen), were both covalently attached to the same latex beads, but not to each other we observed a CD4^+^ T cell-dependent augmented primary, and boosted secondary IgM and IgG anti-PS response. No such effects were observed for beads linked to PS alone or with MSA beads mixed with soluble PS. These responses were enhanced by, but did not require TLR stimulation. These results provided a potential mechanism, i.e., protein aggregation/particulation for the induction of responses to self-proteins normally unable to induce specific T cell or antibody responses. Thus, measures to minimize aggregation of proteins used for therapeutic purposes may lead to a reduction in elicitation of neutralizing or pathogenic antibodies. These data further confirmed our earlier demonstration using 1-µm beads with associated PS and a *foreign* protein (50) that *non-covalent* association of protein and PS was sufficient to elicit T cell-dependent anti-PS responses. The simple association of PS and a foreign protein to a biocompatible particulate substrate might serve as a more cost-effective alternative to the use of PS–protein conjugate vaccines in which the antigens require covalent linkage, especially in developing countries where financial cost may be a limiting factor for widespread usage (51).

## PS Expressed as a Soluble PS–Protein Conjugate Versus the Same PS Expressed by an Intact Bacterium Elicits PS-Specific Antibody Responses from Distinct B Cell Subsets and with Distinct Idiotypes

Parenteral injection of particulate, in contrast to soluble, antigens results in their initial and prolonged concentration within the splenic marginal zone where they come into extended contact with marginal zone B (MZB) cells (52, 53). MZB cells, along with B-1 B cells play a major role in eliciting anti-PS responses (54). Thus, we wished to determine whether MZB cells mediated anti-PS responses to PS-expressing intact bacteria and whether or not this was also true for soluble PS–protein conjugates [the IgG anti-PS responses in both cases were shown to be CD4^+^ T cell-dependent (55, 56)]. For this purpose we utilized Lsc^−/−^ mice. The function of the Lsc protein is to attenuate Gα12/13-mediated G protein-coupled receptor signaling with subsequent activation of RhoA signaling (57). Mice genetically deficient in Lsc (Lsc^−/−^) exhibit a marked defect in MZB migration from the marginal zone following immunization, precluding MZB interaction with CD4^+^ T cells (58). Lsc acts selectively on MZB cells (58, 59).

Lsc^−/−^ mice were immunized and boosted i.p. with intact, inactivated *S. pneumoniae* expressing the type 14 capsular PS or with a soluble conjugate of type 14 PS and the *S. pneumoniae*-derived cell wall protein, pneumococcal surface protein A. Lsc^−/−^ mice exhibited a nearly complete abrogation in the primary and secondary IgG anti-PS responses to intact *S. pneumoniae*, whereas no effects were observed on the same IgG anti-PS response to the soluble PS–protein conjugate (1, 60). In contrast, neither the T cell-independent IgM anti-PS responses to *S. pneumoniae* nor the T cell-dependent IgG anti-protein responses to *S. pneumoniae* or soluble PS–protein conjugate were affected in Lsc^−/−^ relative to control mice. Thus, these data strongly suggested that particulation of associated PS and protein selectively recruited MZB cells to induce a T cell-dependent IgG anti-PS response. This was further supported by our observation that the IgG anti-PS response to a soluble PS–protein conjugate became completely dependent on MZB cells when the conjugate was adsorbed to the surface of an intact *S. pneumoniae* that lacked *natural* expression of both the relevant PS and protein (1).

The selective utilization of MZB cells for the IgG anti-type 14 PS response to intact *S. pneumoniae* was reflected in the observation that the majority of the elicited PS-specific IgG expressed a dominant idiotype, designated 44.1-Id that was not observed when using a soluble conjugate of type 14 PS and protein (61). The idiotype of an antibody is defined as the epitope(s) within the variable region that uniquely defines the specificity of the antibody for its cognate antigen. Of note, attachment of the soluble conjugate to 1-µm latex beads or to the surface of an intact *S. pneumoniae* lacking the relevant PS and protein referred to earlier, resulted in a switch to significant 44.1-Id expression in the elicited IgG anti-PS response. Usage of the 44.1-Id was linked to the *Igh*^a^, but not *Igh*^b^, allotype. These results indicated that different antigenic forms of the same capsular PS can recruit distinct B cell clones expressing characteristic idiotypes under genetic control, and strongly suggested that the 44.1-Id is derived from MZB cells.

## Antibody Responses to Soluble Antigens Involve Distinct APCs Relative to Antigens Expressed by Intact Bacteria

Little is known regarding the specific APCs that initiate T cell activation during T cell-dependent (TD) antibody responses to soluble antigens versus complex particulate antigens, such as inactivated, intact extracellular bacteria. Of note, aluminum salts (“alum”) are often used as adjuvants when immunizing with soluble antigens in various experimental systems, and are themselves particulate. However, antigen adsorbed to alum does not behave as a particulate antigen (1). Thus, DC exposed to alum-adsorbed antigen exhibited facilitated antigen uptake, but did not internalize the alum particles themselves (62).

Dendritic cells, monocytes (and monocyte-derived cells), and macrophages, all of which can serve as APCs, are members of the mononuclear phagocyte system that can be distinguished phenotypically (63, 64). Collectively, they play dominant roles as APCs for CD4^+^ T cells (63). Mouse DC within the spleen are further divided into conventional (classical) (c)DC [either CD8α^+^ or CD11b^+^] and plasmacytoid DC (63, 65–67). Although DC are efficient in uptake of soluble antigens, they also exhibit phagocytic activity. Mouse monocytes are classified as Ly6C^hi^ (“classical monocytes”) and Ly6C^lo^ (“non-classical monocytes”) (63, 68). Ly6C^hi^ monocytes, in particular can internalize and transport antigen to secondary lymphoid organs such as the spleen, where they mature into APCs capable of activating naïve T cells. They are then referred to, generally as monocyte-derived cells (63, 69, 70). Monocyte-derived cells appear to be especially efficient in capturing intact bacteria (71). Macrophages are highly efficient at phagocytosis and play a major role in clearing senescent and apoptotic cells, cellular debris, and pathogens, but are also capable of acting as APC to activate T cells (72). In mouse spleen, macrophages are further divided into red pulp macrophages (73), marginal zone macrophages, and marginal metallophilic macrophages, the latter two located within the MZ (52).

In light of the observation that uptake of intact bacteria and soluble antigens by APCs are skewed toward phagocytosis versus endocytosis or pinocytosis, respectively, we predicted that injection into mice of clodronate-containing liposomes (CL) (74, 75), which are internalized and toxic to highly phagocytic cells, would inhibit CD4^+^ T cell-dependent IgG responses to antigens expressed by intact bacteria but not isolated soluble antigens. Splenic macrophages and monocytes (and monocyte-derived cells), but not conventional DCs or neutrophils, were depleted by i.v. injection of CL (76). Surprisingly, injection of CL markedly inhibited protein-specific IgG responses to a soluble PS–OVA conjugate or OVA alone, as well as to intact, inactivated *S. pneumoniae*. In both instances, CL-mediated inhibition of protein-specific IgG responses was associated with a significant reduction in the formation of germinal centers and the differentiation of CD4^+^ T cells into germinal center T follicular helper cells. However, CL injection which largely abrogated the proliferative response of adoptively transferred OVA peptide-specific transgenic CD4^+^ T cells in response to immunization with *S. pneumoniae* expressing OVA peptide, did not inhibit T cell proliferation in response to soluble PS–OVA or OVA alone. In this regard, monocyte-derived cells depleted by CL, internalized *S. pneumoniae in vivo*, whereas in contrast CD11c^low^ DCs, unaffected by CL injection, internalized soluble OVA. *Ex vivo* isolation and coculture of these respective APCs from *S. pneumoniae*- or OVA-immunized mice with OVA-specific T cells, in the absence of exogenous antigen, demonstrated their selective ability to induce T cell activation. These data provided strong support to the notion that distinct APCs initiate CD4^+^ T cell activation in response to antigen expressed by intact bacteria versus antigen in soluble form. However, CL-sensitive cells appear necessary for the subsequent IgG responses to both forms of antigen (76).

These studies using CL are consistent with earlier studies demonstrating a significant CL-mediated reduction in TNP-specific IgG antibody-forming cells following i.v. immunization with other micron-sized, particulate antigens including TNP-sheep red blood cells (77) or TNP-*Lactobacillus acidophilus* (78), or reduction in serum titers of IgG anti-human serum albumin in response to liposome-associated human serum albumin (79). However, these studies provided no mechanistic basis for these observations. Similarly, i.p. injection of CL resulted in a marked inhibition in priming of CD4^+^ T cells, including IFN-γ^+^ T cells, following i.p. infection with live *Salmonella typhimurium* that was associated with a reduced accumulation of monocyte-derived cells in the spleen (80). However, in contrast to our findings, CL had no effect on the *S. typhimurium*-induced IgG2a plasma blast response and both monocyte-derived cells and conventional DC from *S. typhimurium*-infected mice could activate *S. typhimurium*-specific CD4^+^ T cells *ex vivo*, in the absence of exogenous antigen (80). The use of a live Gram-negative bacterium in this former study, as opposed to a Gram-positive, heat-killed bacterium used in this study, may potentially underlie the observed differences. Of note, i.v. injection of CL failed to inhibit humoral immune responses to smaller, nanometer-sized particles (i.e., inactivated rabies virus or immune-stimulating complexes containing rabies virus antigens) immunized *via* the i.v. route (81). Collectively, these data add further support to the notion that antigens in particulate form have distinct immunologic properties relative to soluble antigens.

## Conclusion and Future Directions

Antigens expressed in particulate/aggregated form exhibit distinct immunologic properties relative to corresponding antigens in soluble form. Cells with high phagocytic activity selectively internalize particulate antigens and do so with relatively high efficiency. Antigen within the particle is displayed in multiple copies facilitating high avidity multivalent B cell cross-linking resulting in higher and sustained levels of B cell activation and antigen internalization for presentation to CD4^+^ T cells. This promotes higher antibody responses to foreign proteins but also a higher likelihood of generating autoantibody-secreting cells. Antigen particulation also allows for coexpression of adjuvant and cell targeting moieties for more efficient and/or targeted immune responses. Particulation itself may further activate the inflammasome and provide intrinsic adjuvant activity. Finally, particles may localize to the splenic marginal zone that may facilitate engagement of MZB that express specialized functional properties. An understanding of the unique immunologic properties of antigens in particulate form should guide future design of vaccines and protein therapeutics.

The following unanswered questions merit further study: (1) how do conventional B cells extract and present antigens from intact bacteria or protozoans in light of their inability to phagocytose particles of ≥1 μm size, (2) what is the significance of the differential usage of select APCs in response to soluble versus particulate antigens on the subsequent nature of the immune response, (3) what is the mechanism by which particulate or aggregated antigens break immunologic tolerance, (4) what are the precise features (e.g., size, composition, organization) of particulate antigens that lead to optimal immune responses, and (5) can directing antigen to MZB cells through particulation be exploited clinically to alter the quantity or quality of the immune response.

## Author Contributions

The author confirms being the sole contributor of this work and approved it for publication.

## Disclaimer

The opinions expressed herein are those of the author, and are not necessarily representative of those of the Uniformed Services University of the Health Sciences (USUHS), the Department of Defense (DOD), or the United States Army, Navy, or Air Force.

## Conflict of Interest Statement

The author declares that the research was conducted in the absence of any commercial or financial relationships that could be construed as a potential conflict of interest.
